# Juvenile Nasopharyngeal Angiofibroma: An Aberrant Case Report

**DOI:** 10.7759/cureus.24350

**Published:** 2022-04-21

**Authors:** Swaragandha Jadhav, Sandeep Khandaitkar, Kajal Mitra, Shyam Chaudhari, Avinash P Dhok

**Affiliations:** 1 Radiodaignosis and Imaging, NKP Salve Institute of Medical Sciences and Research Center, Nagpur, IND; 2 Oral and Maxillofacial Surgery, VSPM's Dental College and Research Center, Nagpur, IND; 3 Radiodiagnosis and Imaging, NKP Salve Institute of Medical Sciences and Research Center, Nagpur, IND; 4 Cardiovascular & Respiratory Physiotherapy, VSPM's College of Physiotherapy, Nagpur, IND

**Keywords:** case report, cect, aggressive, benign, highly vascular, juvenile nasopharyngeal angiofibroma

## Abstract

Juvenile nasopharyngeal angiofibroma (JNA) is a very uncommon condition. We are presenting a case of a teenage boy with painless nasal blockage and fullness of bilateral ears for two to three months. On nasal endoscopy, a firm proliferative mass obstructing the right nasal cavity was noted. It was bleeding on touch. On contrast-enhanced computed tomography of the paranasal sinuses (CECT PNS), a diagnosis of the JNA was made based on the evidence of bony erosion and intense post-contrast enhancement. He was pre-operatively locally embolized followed by surgical resection. On follow-up, the patient was stable with no signs of recurrence.

## Introduction

Juvenile nasopharyngeal angiofibroma (JNA) is a very uncommon, benign, locally aggressive, and highly vascular tumor affecting exclusively young males. It is a life-threatening condition with an incidence of less than 0.5% [1]. A biopsy is contraindicated due to the high vascularity of the tumor [2]. Multiplanar imaging modalities such as contrast-enhanced computed tomography (CECT), magnetic resonance imaging (MRI) of the paranasal sinuses (PNS), and angiography are used to make the diagnosis. These imaging methods aid in determining the tumor bulk, pre-operative feeder vascular embolization, and therapy planning [3].

## Case presentation

We are reporting a teenage boy who came to the Department of Otolaryngology, complaining about the fullness of bilateral ears associated with decreased hearing for three months and painless nasal blockage for two months. The patient was pale on general examination. Systemic examination was within normal limits. Only hemoglobin was reduced. It was 9.5 g/dL, the rest of all laboratory investigations were within normal limits. Mantoux test was negative. On nasal endoscopy, proliferative mass obstructing the right nasal cavity was noted. It was firm in consistency and bled on touch. On posterior rhinoscopy, a dirty white proliferating lesion was seen in the canal crossing the midline.

**Figure 1:**
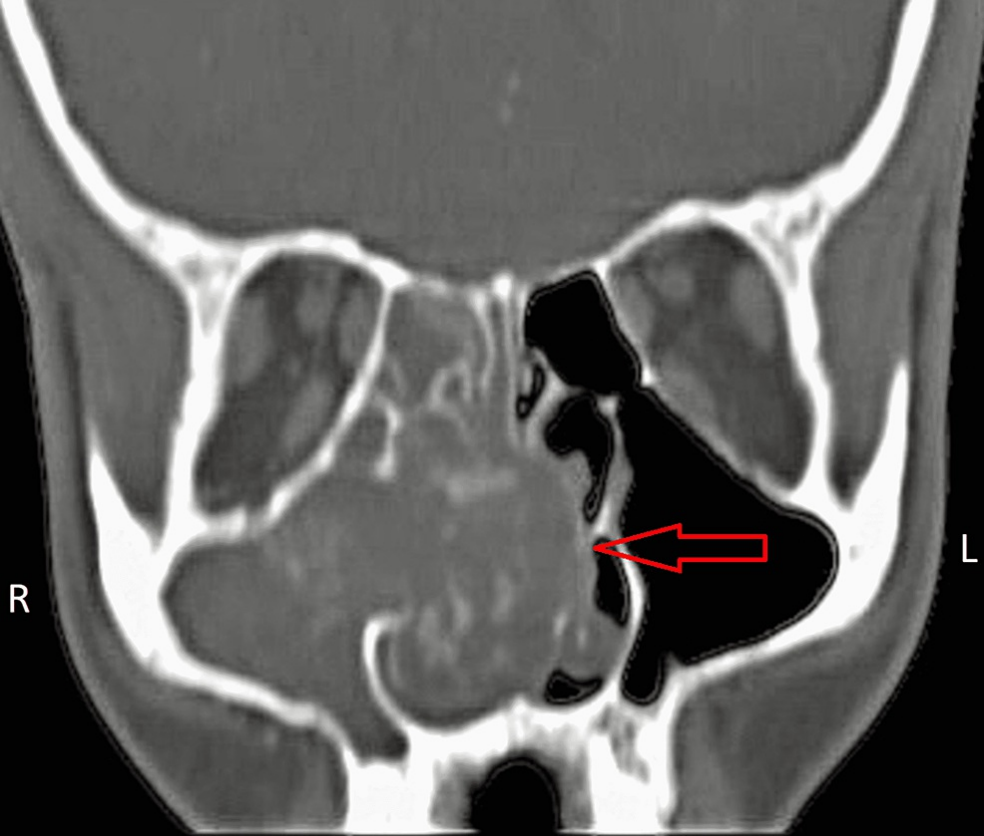
Coronal plane CT-PNS image showing, soft tissue density lesion completely occluding right maxillary sinus, entering the right nasal cavity through the widened ostium with mass effect on the nasal septum resulting in deviated nasal septum with convexity toward the left side causing partial obstruction of the left nasal cavity and atrophy of the corresponding middle turbinate due to compression. PNS - paranasal sinuses

**Figure 2:**
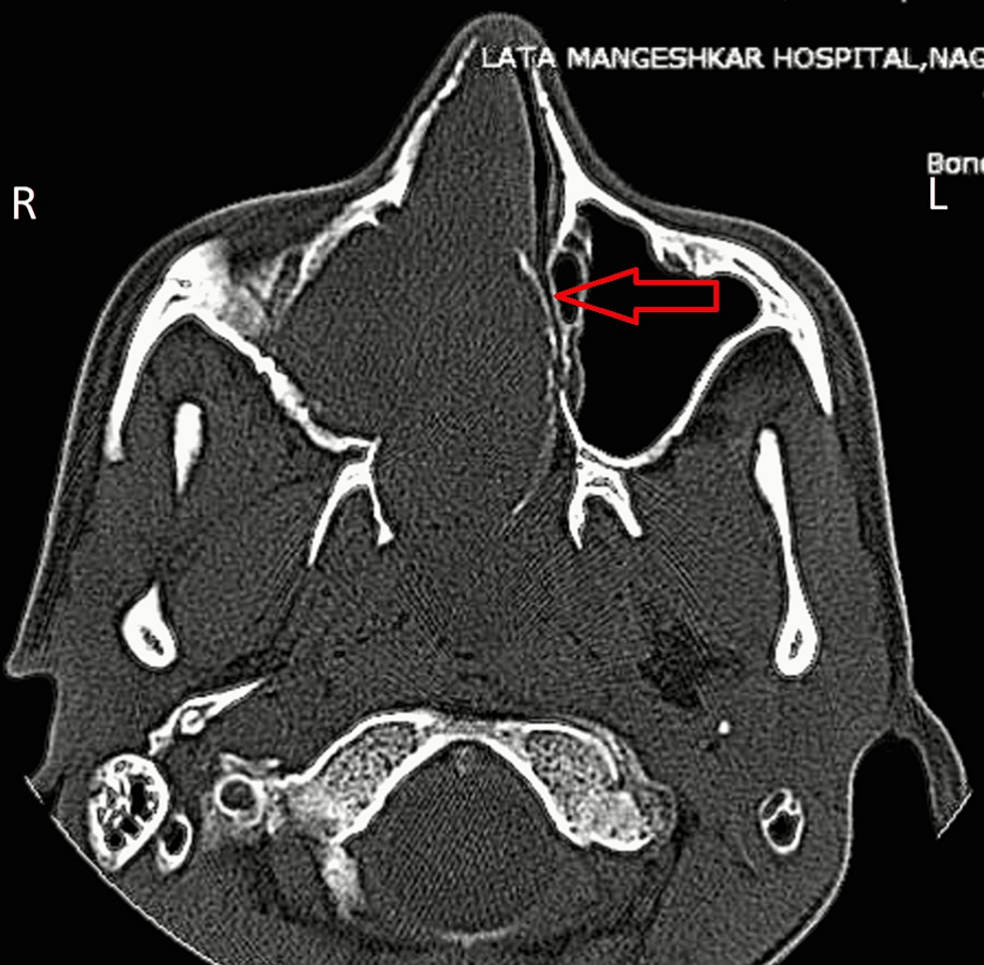
Axial plane of CT-PNS image showing, soft tissue density lesion completely occluding right maxillary sinus, extending anteriorly into the right nasal cavity through the widened ostium with mass effect on the nasal septum resulting in deviated nasal septum with convexity toward the left side and atrophy of the corresponding middle turbinate due to compression. The absence of medial wall of the right maxillary sinus with thinning of the medial pterygoid plate on the right side suggestive of bony involvement. PNS - paranasal sinuses

The patient was advised of the CECT PNS in our department for further evaluation. CECT PNS reveals a well-defined soft tissue density lesion present in the maxillary sinus on the right side and completely occluding it. The lesion was entering the right nasal cavity through the widened ostium. It was extended posteriorly into the nasopharyngeal airway. Posteriorly, the lesion was crossing the midline and causing near-total obliteration of the nasal airways. The lesion was causing a mass effect on the nasal septum resulting in the deviated nasal septum (DNS) with convexity toward the left side and atrophy of the corresponding middle turbinate due to compression. There was also partial obstruction of the left nasal cavity due to DNS (Figures 1, 2).

**Figure 3:**
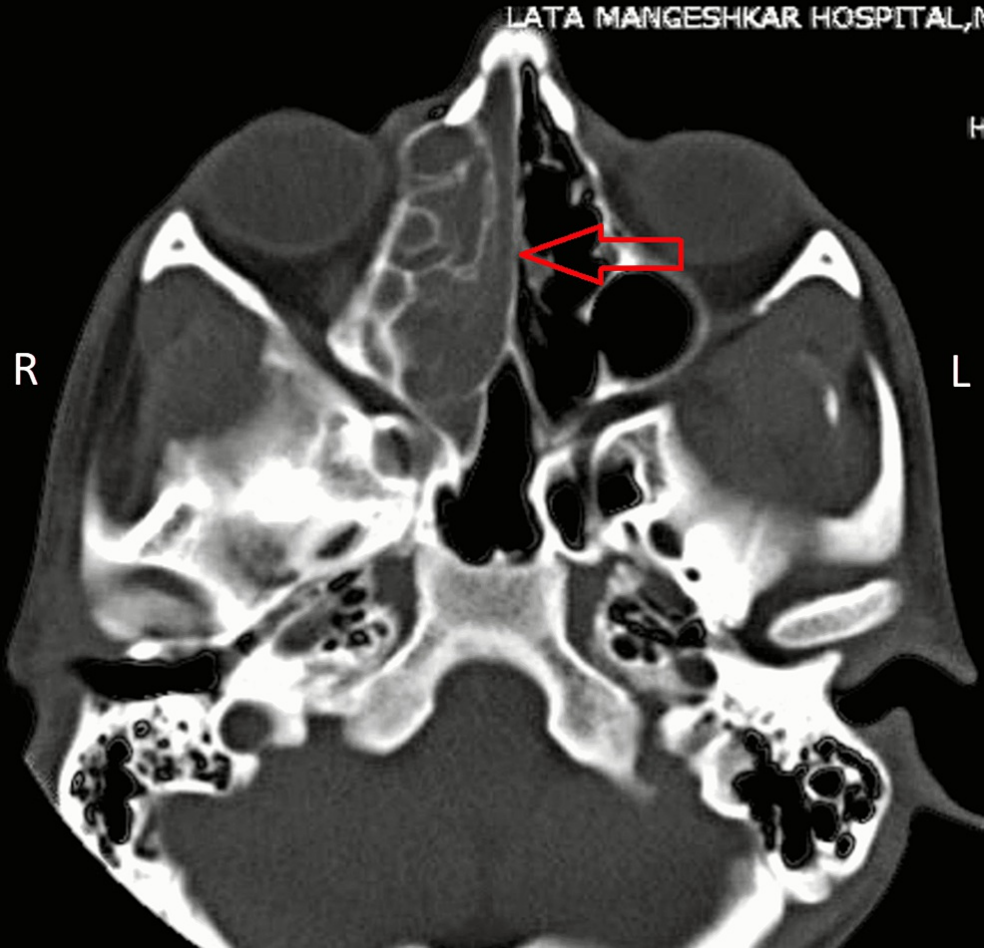
Axial plane CT-PNS image showing soft tissue density lesion completely occluding right ethmoid sinus. PNS - paranasal sinuses

**Figure 4:**
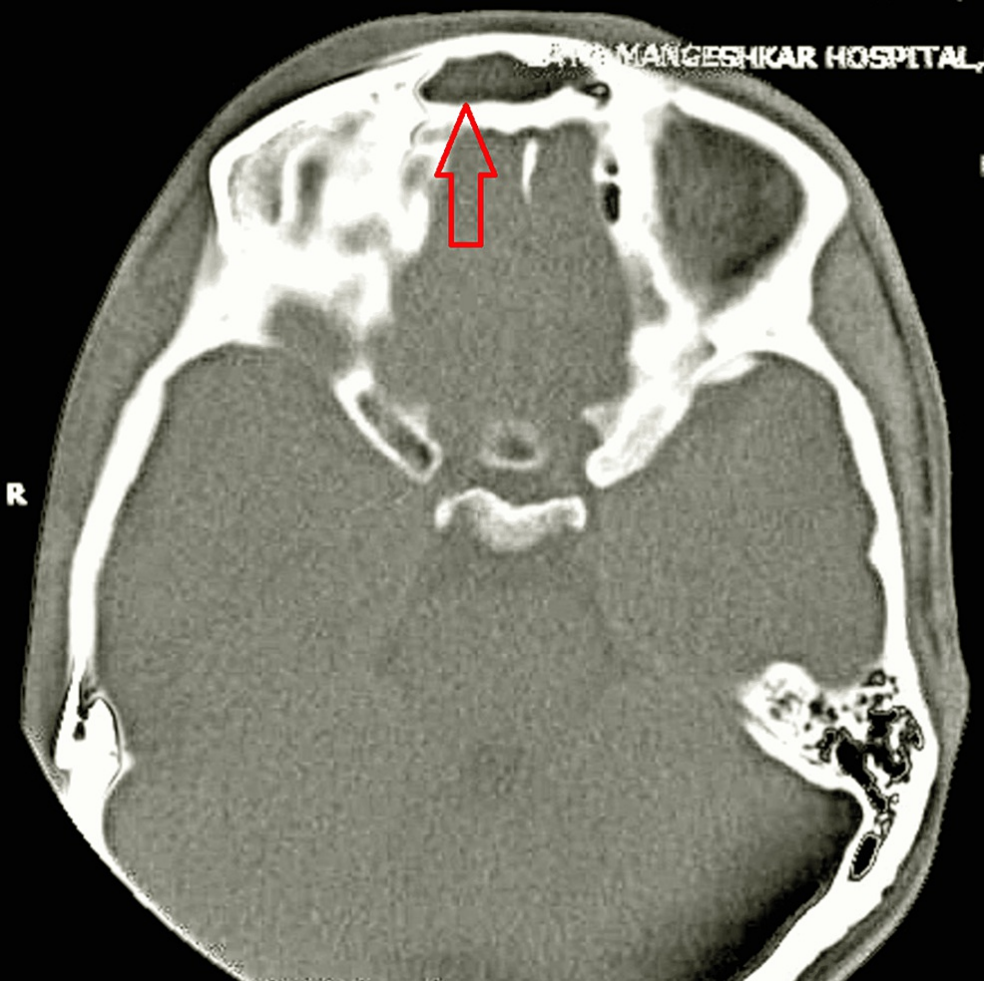
Axial plane CT-PNS image showing soft tissue density lesion completely occluding frontal sinus. PNS - paranasal sinuses

This soft tissue attenuation mass was involving the ipsilateral ethmoidal sinus and it was extending to the frontal sinus (Figures 3, 4).

**Figure 5:**
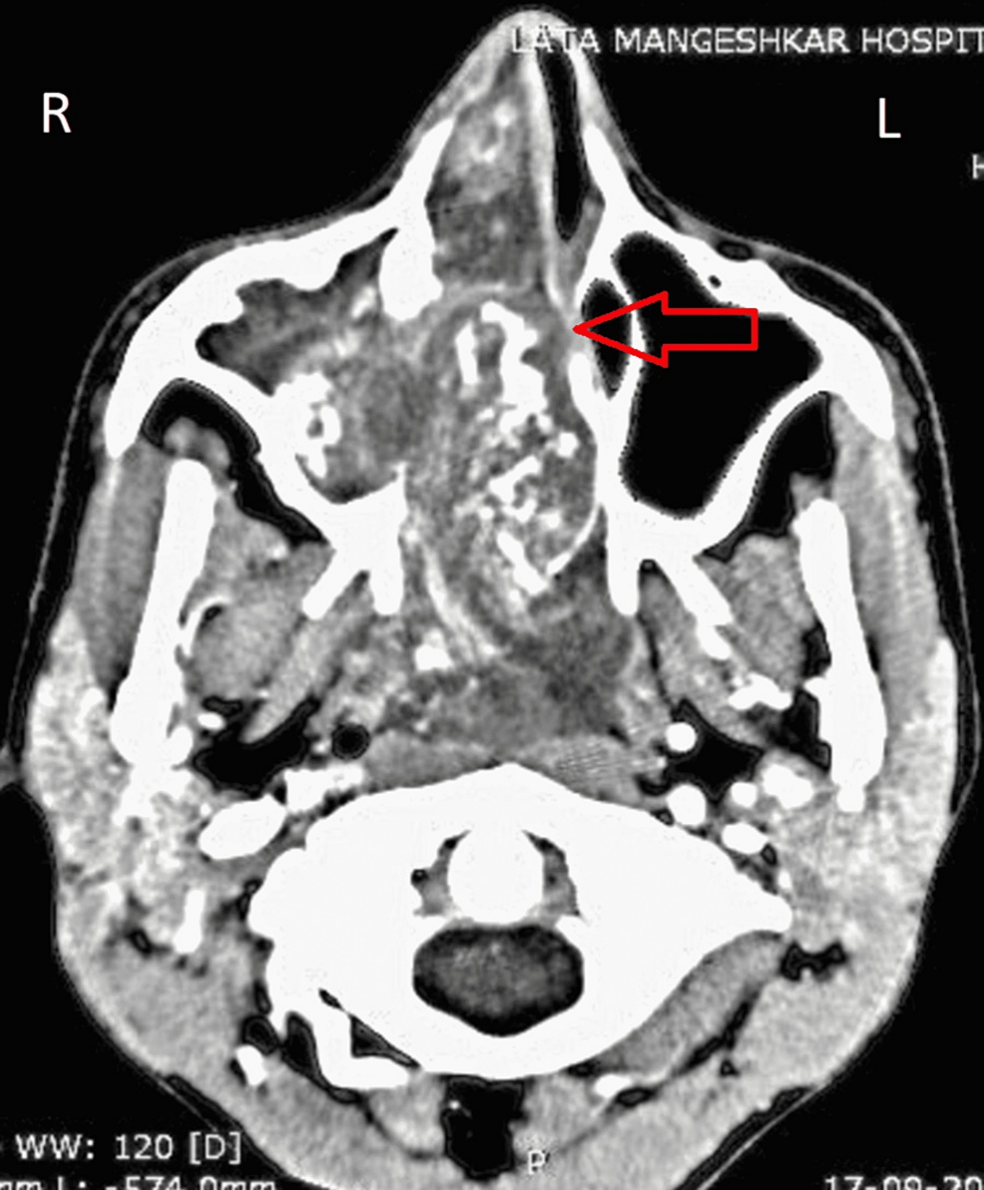
Post-contrast axial plane CT-PNS image showing heterogeneous intense enhancement.

Evidence of bony remodeling was present. The osteomeatal complex on the right side was widened measuring 1.6 centimeter (cm). The lesion was reaching the sphenopalatine foramen but no extension into the pterygopalatine fossa was noted. The right inferior orbital fissure appeared normal. Post-contrast Axial CT-PNS images showing, heterogeneous intense enhancement (Figure 5).

It demonstrated heterogeneously intense amplification in a contrast study. There was thinning of the upper medial pterygoid plate on the right side suggestive of bony involvement. These imaging findings favor the locally aggressive lesion, the possibility of JNA. Nasopharyngeal polyp occurs in the middle age group, a benign condition that never bleeds on touch. Nasopharyngeal carcinoma is more prevalent in the older age population and the site of origin is Fossa of Rosenmuller whereas the current clinical and radiological findings ruled out the above two conditions, i.e., adolescent age group, bleeding on touch, aggressive behavior in the form of bony erosion and high vascularity, confirming the diagnosis of JNA.

The patient was given nasal decongestant, Xylomethazoline for five days, intravenous ceftriaxone 1 g twice daily for three days. Preoperative embolization was done 24 hours before surgery using polyvinyl alcohol followed by debulking surgery. Tumor mass was resected, eroded bony parts were removed. Debridement was done with functional endoscopic sinus surgery. Nasal packing was done for two days to prevent nasal bleeding and alkaline nasal douches were done to remove the nasal crust. The patient was discharged after seven days and advised intravenous ceftriaxone 1 g twice daily for five days. On follow up, healthy nasal mucosa was seen and no residual growth was noted.

## Discussion

JNA is a very uncommon, benign, locally aggressive, and highly vascular tumor. It affects males exclusively from the age group of 5-25 years as the tumor expresses androgen and testosterone receptors only and lacks estrogen receptors [4]. It originates from the sphenopalatine foramen and spreads locally. The patient often presents with complaints of nasal blockage and epistaxis. The patient may experience facial swelling, oto-mastoiditis, anosmia, and dacryocystitis. Bony erosion is also most common in the JNA. On CT scan non-capsulated, soft tissue density mass is usually seen originating from sphenopalatine foramen. Post-contrast study shows enhancement, suggesting the vascularity of the tumor. MRI is the best mode of evaluating soft tissue masses. It appears as heterogeneous altered signal intensity mass on T1 WI and T2 WI. Flow voids are seen on MRI indicating enlarged tumor vessels. Angiography is useful in pre-operatively embolization to find out major feeding vessels. Preoperative embolization is usually performed between 24 and 72 hours before surgery [5]. In correspondence with the cases reported in the literature with the findings of nasal blockage, epistaxis, facial swelling, oto-mastoiditis, anosmia, and dacryocystitis, the teenage boy in the present case had complained about nasal blockage and ear fullness only. In the present case, nasopharyngeal polyp and nasopharyngeal carcinoma were ruled out, due to clinical and aggressive radiological findings. Based on the age, nasophryngeal polyp occurs in middle age and nasopharyngeal carcinoma in older ones whereas JNA is in the adolescent age group. In 1999, Lloyd et al. after investigating 72 patients on CT concluded about specific radiological features of JNA. These features were a soft tissue mass in the nasopharynx, pterygopalatine fossa, and erosion of the posterior osseous margin of the sphenopalatine foramen [6]. He suggested that these three findings on CT should be diagnosed as JNA. These findings hold true in our case scenario.

In 2019, Amran and Bahar described the significance of preoperative embolization with polyvinyl alcohol in highly vascular tumors, such as nasopharyngeal angiofibroma. This procedure can reduce blood loss during the resection of the tumor [7]. Hence, preoperative embolization of the feeder's vessel was done with polyvinyl alcohol in our case.

## Conclusions

This case report reveals an extremely rare case of JNA presented with painless nasal blockage and fullness of bilateral ears for two to three months. It was diagnosed radiologically as aggressive behavior in the form of bony erosion and high vascularity. This case was treated pre-operatively with embolization followed by surgical resection. On follow-up, there was no evidence of recurrence noted. The present case highlights the importance of considering JNA in the differential diagnosis of cases reporting only painless nasal blockage and ear fullness with the view to avoid biopsy to prevent fatal bleeding. This case report adds to the growing knowledge of the significance of diagnosis making in suspected cases of painless nasal blockage, the importance of effective utilization of diagnostic aid in the form of CECT PNS, and mandatory preoperative embolization to identify the feeding vessel as a management tool for JNA.
